# The Salient Aroma Hypothesis: host plant specialization is linked with plant volatile availability in Lepidoptera

**DOI:** 10.1098/rspb.2024.2426

**Published:** 2025-03-12

**Authors:** Po-An Lin, Wei-Ping Chan, Liming Cai, Yun Hsiao, Even Dankowicz, Kadeem J. Gilbert, Naomi E. Pierce, Gary Felton

**Affiliations:** ^1^ Department of Entomology, National Taiwan University, Taipei, Taiwan; ^2^ Department of Organismic and Evolutionary Biology, Harvard University, Cambridge, MA, USA; ^3^ Department of Integrative Biology, The University of Texas at Austin, Austin, TX, USA; ^4^ Institute of Ecology and Evolutionary Biology, National Taiwan University, Taipei, Taiwan; ^5^ Department of Plant Biology, Michigan State University, W K Kellogg Biological Station, Hickory Corners, MI, USA; ^6^ Museum of Comparative Zoology, Harvard University, Cambridge, MA, USA; ^7^ Department of Entomology, The Pennsylvania State University, University Park, PA, USA

**Keywords:** volatile organic compounds, specialization, host range, diet breadth, diel activity, information availability

## Abstract

Host plant use in Lepidoptera has been a primary focus in studies of ecological specialization, and multiple factors are likely to be involved in shaping the evolution of diet breadth. Here, we first describe the Salient Aroma Hypothesis, suggesting that the availability of chemical information, particularly host-associated aromas, plays a critical role in shaping dietary specialization. According to the Salient Aroma Hypothesis, herbivores active during periods when chemical information is abundant, particularly during the daytime hours when plant aromas are hypothesized to be more prevalent, are more likely to evolve specialized diets. First, with meta-analysis, we show that plants release more diverse and abundant volatile compounds during daylight hours, increasing the availability of chemical information. We found that diurnal Lepidoptera tend to have specialized diets, while nocturnal species are more generalized, consistent with the prediction of the Salient Aroma Hypothesis. We further observed that morphological differences in the antennae of female Lepidoptera are correlated with variation in diet breadth and diel activity patterns, indirectly supporting the Salient Aroma Hypothesis. While multiple factors influence host plant specialization, the Salient Aroma Hypothesis offers a useful framework linking chemical information availability (e.g. plant volatiles) and ecological specialization.

## 1. Introduction

Ecological specialization is a widespread phenomenon in biological systems [1,2]. Herbivorous insects are notable for specializing on particular host plants, with less than 10% of herbivores feeding on more than three host plant families [3]. Most herbivores specialize on specific groups of plants [4], although exceptional cases of diet generalists also occur [5]. The relationship between lepidopteran herbivores and their host plants is one of the most thoroughly studied examples of ecological specialization, and research on this topic has spanned multiple contexts, including but not limited to coevolutionary arms races [6,7], evolutionary dynamics [8,9], genetic architecture [10], and geographic range and voltinism [11]. Despite considerable advances in our understanding of factors driving host specialization, explanations for the broad pattern of host specificity observed in phytophagous insects remain unclear [12].

Host range of most Lepidoptera is governed by adult females laying eggs on appropriate host plants, as well as the ability of larvae to overcome plant defences [13]. In seeking oviposition sites, females employ a combination of hierarchical and concurrent cues. Visual cues may initially guide them towards vegetation [14–16], while gustatory cues refine the process through the detection of specific plant chemicals [17]. Notably, chemical information, particularly olfactory cues by plant volatiles, is often used for mid-range foraging, for both floral and vegetative tissues [18–21], serving as signals for host plant selection by both butterflies [22] and moths [23,24]. The diversity and quantity of these volatile cues are important for insect herbivores to locate their host plants [25–28]. Changes in abundance and diversity of volatiles affect the host-searching efficiency and potential specialization of insect herbivores [29–31]. Recent advances in chemical ecology, emphasizing the chemical information (e.g. plant volatiles) for host-searching by insect herbivores, form the basis for the Salient Aroma Hypothesis (SAH).

The synthesis and emission of plant volatiles, both defensive and non-defensive, are tied to photosynthesis and stomatal activity, and thus imply a likely circadian pattern [32]. Plants appear to emit higher amounts and more diverse blends of volatiles during the day than at night [33–35], yet a systematic investigation across diverse plant taxa is lacking.

Like plants, Lepidoptera tend to display a strong diel activity preference usually by the time they mate and forage [36]. Since foraging behaviour requires host-associated chemical information that assists decision-making, it seems likely that the diel cycle of plant volatile emissions influences the foraging choices of ovipositing herbivores. Lepidoptera that are not strictly nocturnal, which we refer to here as light-active species, are likely to encounter more volatile cues during daylight (or photophase) hours than at night (or scotophase). The enhanced availability of chemical information presumably provides greater opportunities for more precise host searching and identification.

**Figure 1.**
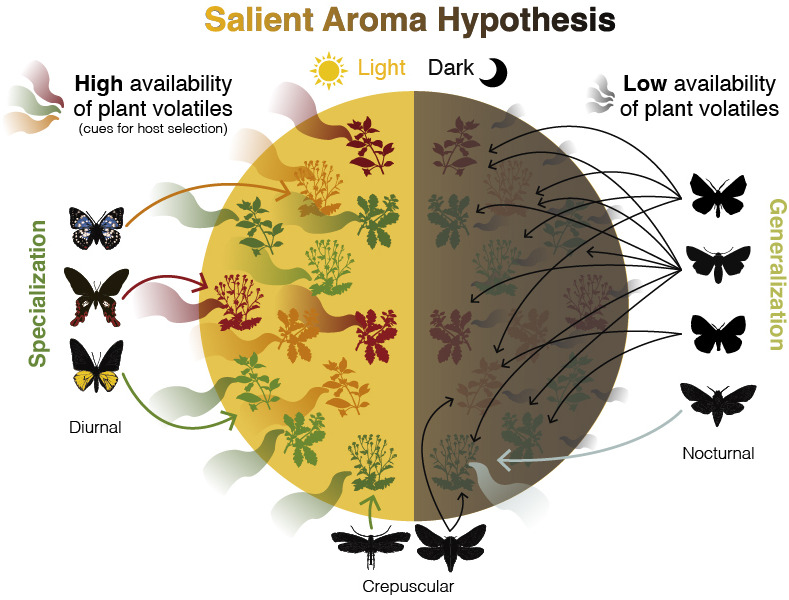
The Salient Aroma Hypothesis. Plants exhibit diel patterns of volatile emission, releasing more volatiles during the photophase than the scotophase, creating temporal variation in chemical information availability. This variation in chemical information may influence herbivore host specialization, with higher availability promoting specialization and lower availability favouring generalization.

Here, we propose the SAH (figure 1) based on the intersection between the daily cycles of plant volatile availability, the daily activity patterns of adult Lepidoptera and their host plant choices. According to this hypothesis, salient volatile cues—those that are significant for herbivores in identifying and locating host plants—may facilitate host plant selection and specialization in Lepidoptera. Thus, the evolution of host plant specialization in Lepidoptera may be influenced by ecological factors, specifically the availability and diversity of ecologically meaningful chemical information, such as plant volatiles.

To detect plant volatiles, herbivorous insects typically possess advanced olfactory abilities [37]. Olfaction is also often correlated with both the number of sensilla [38–40] and the surface area [40,41]. For example, larger antennae have been shown to be correlated with enhanced chemical sensing ability in foraging bumble bee workers [42] and males locating females by their sex pheromones [40,43–45].

In environments where there is an opportunity to utilize specific cues, sensory traits tend to be more elaborate. This occurs whether these cues are abundant or scarce. For example, animals in environments with either high or low light availability can develop complex visual systems [46]. Although much work has been done on how the light environment influences visual traits, less is known about how the chemical environment might influence olfactory traits.

Selection favours investment in sensory organs when detection of scarce, yet present stimuli is critical for survival. For example, the well-developed eyes of nocturnal species active in dim light conditions indicate an increased investment in detecting scarce yet present cues [46], as are the antennae of male moths detecting sex pheromones [43]. In herbivores, the ability to locate hosts is crucial for survival, so the high availability of plant volatiles during the day likely drives the evolution of larger olfactory structures, especially in host-seeking females.

Although the scarcity of volatiles at night may promote the development of more refined olfactory systems to detect these cues, as in the antennae of male moths, it is also possible that scarcity of volatiles can lead to reduced investment in olfactory organs. When the opportunity to use environmental cues is absent, organisms often exhibit a reduction in the corresponding sensory traits. For instance, cave-dwelling fish may lose their eyesight entirely [47]. Similarly, moths in bat-free habitats exhibit reduced auditory sensitivity [48], highlighting how relaxed selective pressures can lead to regressive evolution of sensory traits.

Variation in the availability and diversity of volatiles across time and space is exacerbated due to the transient nature of plant volatiles, which break down quickly in the atmosphere [30,49–55]. The need for specific receptors to detect these volatiles [40,56] and the necessity of detecting specific combinations of these volatiles to make them ecologically meaningful for herbivores [26] suggest that Lepidoptera active during periods with limited plant-associated volatiles might have less opportunity to utilize these plant cues. As a consequence, they may become less chemically receptive, and this could lead to less elaborate olfactory structures compared with those in environments rich in chemical information.

Following this reasoning, we argue that volatile availability (linked to diel activity) likely influences the size and/or sensitivity of Lepidoptera antennae. Given the impracticality of investigating receptor abundance and function across hundreds of species, we focused instead on measuring antennal size as a more feasible proxy for olfactory investment [38,42,57]. We predict that specialist herbivores should have larger antennae than their generalist counterparts due to the increased ecological demand for accurately locating specific host plants. We further hypothesize that species active during the day may have larger antennae given the greater availability of chemical information in daylight. For nocturnal species, two scenarios seem possible. If volatile cues are scarce but important for survival, these species might evolve larger antennae, analogous to the evolution of complex nocturnal eyes or elaborate antennae in male moths seeking females emitting pheromones [43,45]. However, if low availability of plant volatiles significantly limits the opportunity for insects to use these cues in host seeking, this could lead to reduced antennal size, especially in females.

Our analyses apply meta-analytic, phylogenetic and morphological comparative approaches to examine potential links between: (i) diel emission patterns of plant volatiles, (ii) host plant specialization of Lepidoptera, (iii) diel activity preferences of Lepidoptera and (iv) lepidopteran antennal morphology. Specifically, we investigate whether in general, plant volatiles are more abundant and diverse during the daytime than at night. We then examine whether this pattern is correlated with key behavioural and morphological traits associated with host plant specialization in Lepidoptera by assessing the activity patterns of different species, their host breadth, as well as the relative sizes of their antennae.

## 2. Material and methods

### (a) Literature-based characterization of plant volatile emission pattern

We searched Google Scholar for studies on diel plant volatile emissions using keywords such as ‘plant’, ‘biogenic’, ‘volatile’, ‘diel’, ‘circadian’, ‘rhythms’, ‘light or dark’ and ‘day or night’. After applying quality criteria (electronic supplementary material, note S1), we selected 115 references covering 149 cases of distinct plant species or landscapes (electronic supplementary material, table S1). Data were categorized by attributes including: (i) collection location (laboratory or landscape), (ii) plant habit (e.g. conifer, broadleaf tree, shrub, herbaceous plant), (iii) plant family and species, (iv) physical damage (undamaged or herbivory), (v) stress type (non-stressed, biotic, abiotic), (vi) volatile source (leaf, floral, landscape) and (vii) flowering time (day or night). We extracted means, standard deviations (s.d.), or standard errors (s.e.) for daytime and night-time volatile emissions and, where available, recorded the number of volatile compounds reported in the literature (electronic supplementary material, note S1).

### (b) Meta-analysis of the diel volatile emission pattern

Meta-analysis synthesizes findings from multiple studies to identify patterns using effect size measures like Hedges’ *g*, which standardizes mean differences by variance for an unbiased effect size estimate [58,59]. We used the *metacont* function in the R package *meta* [60] to calculate Hedges’ *g*, quantifying differences in daytime versus night-time volatile emissions. A random-effects model (inverse variance method) accounted for variability within and between studies [61].

To assess factors influencing day–night volatile differences, we performed meta-regression, identifying sources of heterogeneity across studies [62]. This analysis covered volatiles from different sources (flowers, leaves, landscapes), plant habits (e.g. conifers, shrubs, herbaceous) and families. Small study effects were evaluated via funnel plots, trim-and-fill analysis (*trimfill* function in *meta*), and the Begg & Mazumdar Test (*metabias* function) [59], ensuring robust results (electronic supplementary material, note S2, figure S1; [60]). We also analysed day–night volatile diversity across 125 cases using a generalized linear model (package *lme4* function *glm*) with a Poisson distribution and log link function [63] predicting the number of identified compounds based on treatment (day or night).

### (c) Lepidoptera phylogeny and diel activity preference

We used the most comprehensive Lepidoptera phylogeny (197 species, 84 families, 34 superfamilies) as our reference tree [64]. Diel preferences were recorded primarily from peer-reviewed sources, supplemented by online records when necessary (electronic supplementary material, table S2). Host plant data were available for 145 species and included in downstream analysis. Although diel activity is a continuous trait [65], it is typically categorized due to limited behavioural data. To account for this, we classified diel activity in two ways: a two-state model (diurnal versus nocturnal) and a four-state model (all-day, crepuscular, diurnal and nocturnal) following Kawahara *et al*. [36]. These categories apply to adult insects, particularly female host-finding behaviours, though detailed records are scarce. To avoid bias, we excluded light trap records, which may misrepresent diel activity, and did not incorporate larval diel activity preferences.

### (d) Lepidoptera host plant diversity and correlated evolution of diel preference and host specificity at plant family and genus levels

For each species in the Lepidoptera phylogeny, we determined host plant preferences based mostly on peer-reviewed publications. For species where peer-reviewed publications were unavailable, we determined the host plants using field guides and databases [66].

To evaluate the correlation between Lepidoptera diel preference and host family specificity, we used a binary phylogenetic generalized linear mixed model (PGLMM), which incorporates phylogenetic relationships and random effects to account for non-independence of data points due to shared ancestry [67]. For host family specificity, species were scored as either specialist (feeding on one plant family) or generalist (feeding on multiple families) [4]. We then used the function *binaryPGLMM* from the R package *ape* [68] to test for the significance of trait correlation under the PGLMM model. We calculated phylogenetic variance (*s*^2^) to measure trait variance attributable to shared ancestry. A significant *s*^2^ indicates similar diel preferences among closely related species [67]. To address the issue of uneven representation across our phylogeny (further elaborated in the discussion), which is skewed towards certain lepidopteran lineages (like Papilionoidea), we implemented random sampling within our dataset to mitigate selection bias. To ensure a balanced representation and reduce potential sampling bias (electronic supplementary material, note S3, figure S2), we conducted subsampling analyses using bootstrapping with 1000 iterations with two distinct random sampling strategies. The first method equalized the number of sampled species across diel activity categories, minimizing bias related to functional comparisons. The second method balanced the representation across superfamilies, preserving phylogenetic diversity (PD) based on Lepidoptera composition [69]. Both approaches yielded results highly consistent with our main analyses (electronic supplementary material, note S3, figure S2) demonstrating the robustness of our findings and providing a framework to address taxonomic biases in similar datasets.

For an in-depth examination of host diversity and diel activity preferences among Lepidoptera, we used phylogenetic metrics to measure richness and the extent of divergence of host plants scored at genus level. Our phylogenetic assessments were anchored to a calibrated phylogeny of seed plants as outlined by Smith & Brown [70]. We pruned the tree to include two species per plant genus whose divergence time represented the crown group age of the genus. For paraphyletic genera, we used a custom Python script to identify the earliest diverging monophyletic clade to set the crown group.

Second, we used the functions *pd*, *ses.mpd* and *ses.mntd* from the R package *picante* to calculate six phylogenetic metrics to measure the richness and divergence of host plants [71]. For host plant richness, the raw number of host plant genera (electronic supplementary material, table S3) and Faith’s PD were used [72]. For host plant divergence, the mean pairwise distance (MPD) and the mean nearest taxon distance (MNTD) [73] were calculated. To further assess specialization, we included the distance-based specialization index as a *Z*-score of MPD or MNTD, which measures deviation from a random expectation using a null model with 200 bootstrap replications [74]. These metrics provided complementary insights into host diversity and specialization across different Lepidoptera species. The complete R script used for these calculations is publicly available on GitHub (https://github.com/lmcai/day_and_night_moths).

Finally, to evaluate the significance of differences in host diversity between species exhibiting different diel activity preferences (both two-state and four-state), we conducted the PGLMM in R package *stats*. To visualize the character states across phylogeny, we reconstructed the ancestral state of diel activity (two states) and host specificity (two states) under the all-rates-different model using the *ace* function in the R package *ape* [68]. This approach enables the estimation of character state evolution while accounting for varying rates of trait change across phylogenetic branches, providing insights into how these traits evolved over time [75].

### (e) Antennal size quantification

Antennal size was quantified using specimens from the Lepidoptera collection of the Museum of Comparative Zoology. We included six male and six female specimens for each species. Ten of the 197 species without adequate replicates were not included (electronic supplementary material, table S4). The dorsal and ventral sides were imaged using a high-resolution Digital Single-Lens Reflex camera (Nikon D800) for each specimen. After background removal and image segmentation in MATLAB R2019b, the estimated area of each antenna was then used as a proxy for its actual size. To take body size variation into account, we measured the length and width of the body and then normalized the antennal size by the body size, which was estimated as an ellipsoid, by taking the residuals after conducting phylogenetic GLMM [76]. This normalization process removes the body size signal while accounting for phylogenetic relationships among species (electronic supplementary material, figure S3). Both left and right antennal sizes were measured. Finally, the antennal size of each species was determined and scored as the median across replicates for each of the two sexes (electronic supplementary material, table S4).

## 3. Results

### (a) Diel emission pattern of plant volatiles

**Figure 2.**
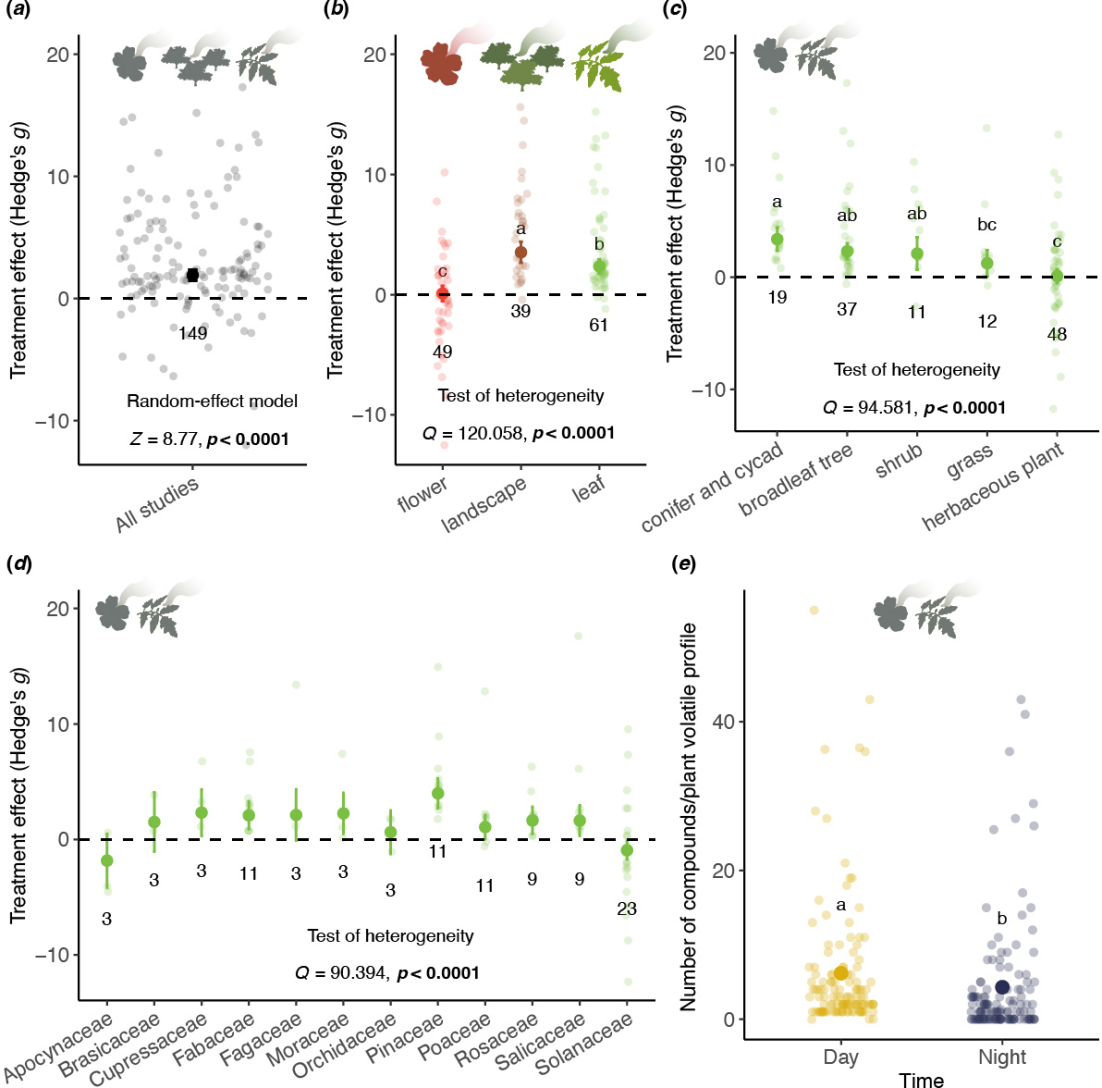
A survey of diel emission patterns of plant volatiles for 65 species representing 57 plant families. This result shows that plants release more volatiles during the day than at night. (*a*) Result of meta-analysis on diel volatile emission patterns from all studies. (*b*–*d*) Results of subgroup analyses on (*b*) volatile type, (*c*) plant habit and (*d*) plant family. Hedges’ *g* indicates the standardized mean differences between daytime and night-time volatile emissions. Numbers below the circles indicate sample size. The letters above the circles indicate significant differences between subgroups. (*e*) Number of different compounds present in the volatile profile of different plants (*n* = 125 cases). Different letters indicate statistical significance (GLM, *p* < 0.05, Tukey HSD). Icons on top of the panels indicate volatile types.

Regardless of growth form or evolutionary history, plants emit significantly higher amounts and more diverse volatiles during the day than at night (figure 2*a*,*b*). The increase in daytime emissions is substantial (Hedges’ *g* = 1.66, *Z* = 8.77, *p* < 0.0001), with volatile types also varying significantly in effect size (*Q* = 120.06, *p* < 0.0001), indicating heterogeneity among studies [77]. Landscape-level studies report the largest day–night differences (Hedges’ *g* = 3.40), with vegetative volatiles showing greater variation (Hedges’ *g* = 2.29) than floral volatiles (Hedges’ *g* = 0.06). Emission patterns also differ by growth habit (figure 2*c*, *Q* = 94.58, *p* < 0.0001). Woody plants, such as gymnosperms (Hedges’ *g* = 3.38) and broadleaf trees (Hedges’ *g* = 2.30), exhibit the highest day–night differences, followed by shrubs (Hedges’ *g* = 2.10), grasses (Hedges’ *g* = 1.24) and herbaceous plants (Hedges’ *g* = 0.09). Significant differences also exist among plant families (figure 2*d*, *Q* = 90.39, *p* < 0.0001). While most families emit more volatiles during the day, Apocynaceae and Solanaceae emit more at night (Hedges’ *g* = −1.83 and −0.94, respectively), aligning with their recruitment of nocturnal pollinators and well-studied floral volatiles (electronic supplementary material, table S5). Night-time volatile collections contain significantly fewer compounds (figure 2*e*, estimate = −0.36, *p* < 0.0001), with diversity higher during the day (6.18 compounds) than at night (4.3 compounds) across 125 cases. Correlation analysis of studies with detailed compound lists (*n* = 24) confirmed a significant association between increased daytime emissions and volatile diversity (Pearson correlation = 0.593, *p* = 0.0022, *t* = 3.46, d.f. = 22). This suggests higher volatile emissions generally correspond to greater chemical diversity.

### (b) Associations between adult diel activity of Lepidoptera and host plant specificity at genus and family levels

We incorporated data for 145 out of the 197 (73.6%) species from the reference Lepidoptera phylogeny to investigate the association between diel activity and host family specificity. Our dataset included 69 light-active (7 diurnal generalists, 49 diurnal specialists, 2 crepuscular specialists and 1 crepuscular generalist, 9 all-active specialists and 1 all-active generalist) and 76 night-active species (37 nocturnal generalists, 39 nocturnal specialists). Given that the existing phylogeny is based on a restricted dataset of exemplars from only 62 out of 126 families and 31 of 46 superfamilies, we also carried out sensitivity analyses (bootstrapping with 1000 iterations) using two distinct random sampling methodologies (electronic supplementary material, note S3, figure S2).

**Figure 3.**
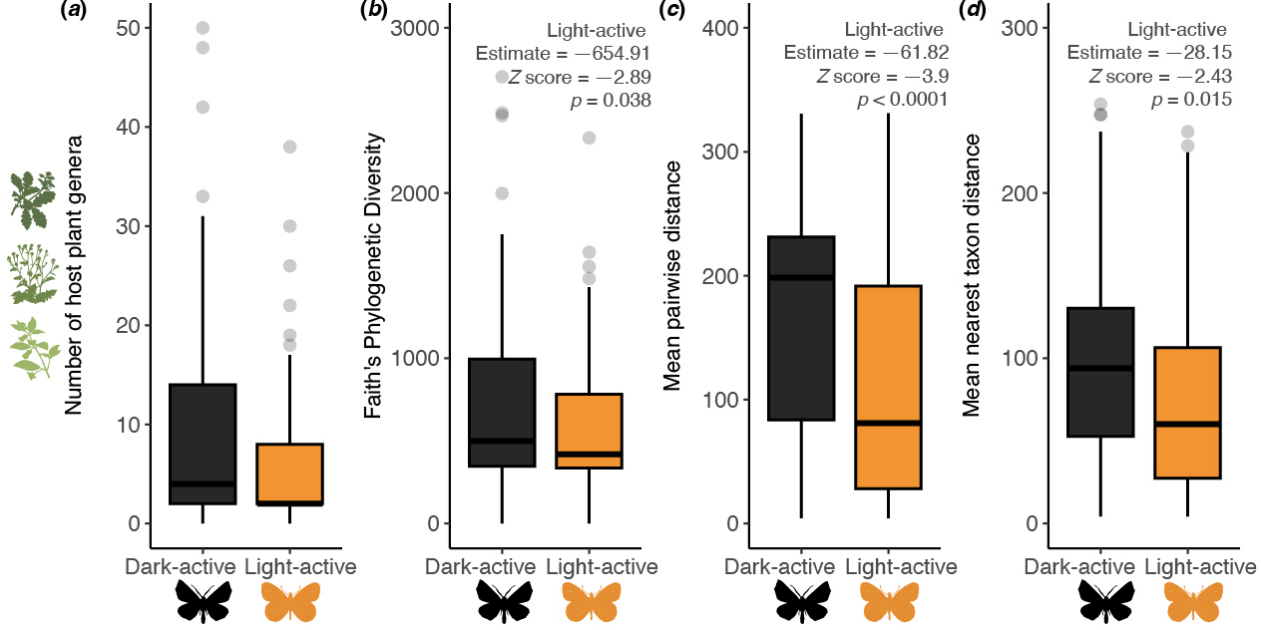
Host plant diversity, measured by the number of host genera consumed, varies with adult diel activity in 136 Lepidoptera species. Species that are light-active for any part of the cycle (diurnal, crepuscular or generalist) are significantly more specialized in host plant choice, both in number and PD, than nocturnal species. (*a*) Host plant genera per Lepidoptera species. (*b*) Faith’s PD, reflecting evolutionary diversity of host plants, is lower in light-active species, indicating reduced phylogenetic breadth in host use. (*c*) MPD, measuring evolutionary divergence among host plants, is lower in light-active species, supporting their specialization. (*d*) MNTD, quantifying evolutionary proximity to the closest related host plant, is also lower, reinforcing their preference for closely related taxa. Statistical results are summarized in PGLMM models (table 1).

To examine the link between lepidopteran diel activity and host plant diversity, we collected genus-level host data for 136 of 197 species (69.0%) in the higher-level Lepidoptera phylogeny (figure 3). The median number of host genera per species is 3.5, with 29.4% feeding on only one genus. Nocturnal species feed on an average of 17.12 plant genera, compared with 7.42 in diurnal species (figure 3*a*). Diurnal lepidopterans have significantly lower host plant PD (figure 3*b*, PGLMM, estimate = −654.91, *Z* = −2.89, *p* = 0.0038, table 1). Pairwise phylogenetic distance analyses confirm that diurnal species also have significantly lower host plant divergence than nocturnal species (figure 3*c*, PGLMM, estimate = −61.82, *Z* = −3.9, *p* < 0.0001; figure 3*d*, PGLMM, estimate = −28.15, *Z* = −2.43, *p* = 0.015, table 1). Expanding analyses to four diel activity states (diurnal, nocturnal, crepuscular and generalist) yielded non-significant results for PD, MPD and MNTD (electronic supplementary material, figure S5 and table S6), likely due to sample size disparities (nocturnal = 76, diurnal = 56, crepuscular = 3 and generalist = 10).

**Table 1.** Results of PGLMM estimates: associations between diel activity preference and host specificity (Faith’s PD, MPD and MNTD). MNTD, mean nearest taxon distance; MPD, mean pairwise distance; PD, phylogenetic diversity; PGLMM, phylogenetic generalized linear mixed model.

	estimate	SE	*Z*-score	*p*‐value
two-level diel activity				
PD				
intercept	1324.55	161.34	8.21	<0.0001
light-active	−654.91	226.38	−2.89	0.0038
MPD				
intercept	166.19	11.31	14.7	<0.0001
light-active	−61.82	15.86	−3.9	<0.0001
MNTD				
intercept	102.47	8.24	12.43	<0.0001
light-active	−28.15	11.56	−2.43	0.015

^a^ ‘Light-active’ species are those classified as diurnal, crepuscular or all-active throughout the day and night.

**Table 2.** PGLMM model estimates: associations between diel activity preference and host specificity (two states). PGLMM, phylogenetic generalized linear mixed model.

	estimate	s.e.	*Z*-score	*p*‐value	*s* ^2^
two-level diel activity					3.018 (*p* < 0.0001)
intercept	0.49	0.88	0.56	0.57	
light-active	1.80	0.46	3.96	<0.0001	

*s*^2^ = phylogenetic signal.

To examine the link between diel activity and host plant specificity, we classified Lepidoptera as light-active or dark-active and as specialists (feeding on a single plant family) or generalists (feeding on multiple families). Light-active species are significantly more likely to be specialists (table 2, PGLMM, estimate = 1.8, *Z* = 3.96, *p* < 0.0001), and diel activity preference shows a strong phylogenetic signal (*s*² = 3.018, *p* < 0.0001). Bootstrapped sensitivity analyses confirmed these results (electronic supplementary material, figure S2, note S3), as did analyses using four diel activity states (electronic supplementary material, tables S6 and S7). Overall, our findings support the hypothesis that nocturnal Lepidoptera are more likely to have generalist diets.

**Figure 4.**
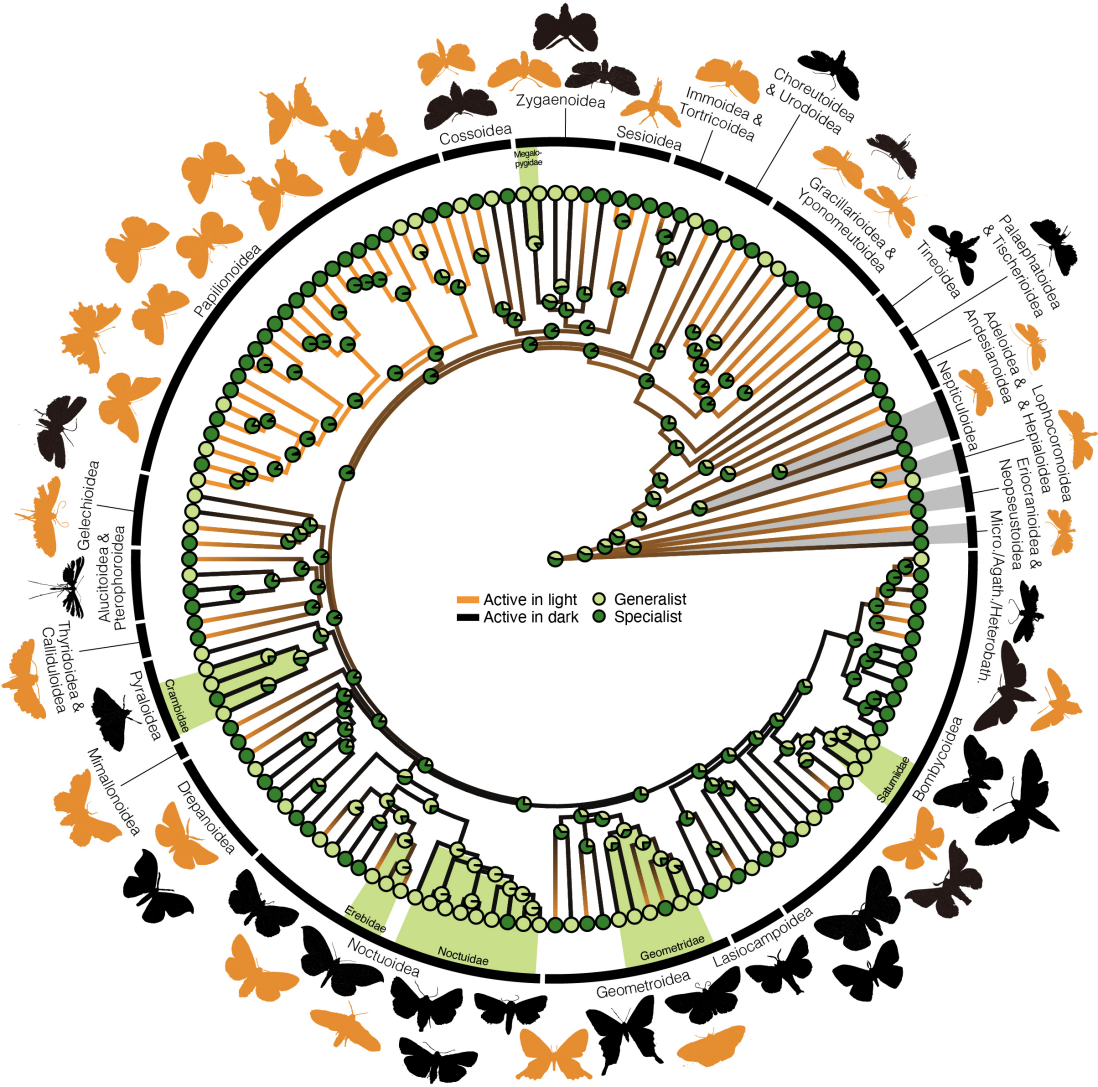
Ancestral state reconstruction suggests that early Lepidoptera were likely diurnally active as adults and host plant specialists as larvae. Circular diagrams at tips and nodes represent host specificity: dark green indicates specialists (single plant family), and light green indicates generalists (multiple families, posterior probability). Adult diel activity is shown by branch colour (posterior density map): yellow/black represent light- and dark-active species, respectively, with transitions marked by colour shifts. Shaded light green areas indicate ancestrally generalist clades, while shaded grey highlights early diverging lineages. Lepidoptera exhibit diverse life histories, including diurnal versus nocturnal activity and generalist versus specialist feeding. Our analysis, however, covers only a small fraction of Lepidoptera species (<0.1%).

Ancestral state reconstruction suggests the earliest Lepidoptera were slightly more likely to be specialists (55.42%) than generalists (44.57%) and light-active (54.27%) rather than dark-active (45.73%). Generalist diets are more common in terminal branches, while most internal nodes are inferred as specialists (67.7% on average). Notably, early-diverging ‘micromoth’ lineages (e.g. Nepticuloidea) show equivocal diet preferences (50.12−65.27% specialists) (figure 4). Clades ancestrally generalist also tend to be ancestrally nocturnal, including Actias–Attacus, Biston–Idaea, Agrotis–Amphipyra and Arctia–Amata (figure 4, electronic supplementary material, figure S4).

### (c) Links between morphological traits, lepidopteran diel activity preferences and host specialization

**Figure 5.**
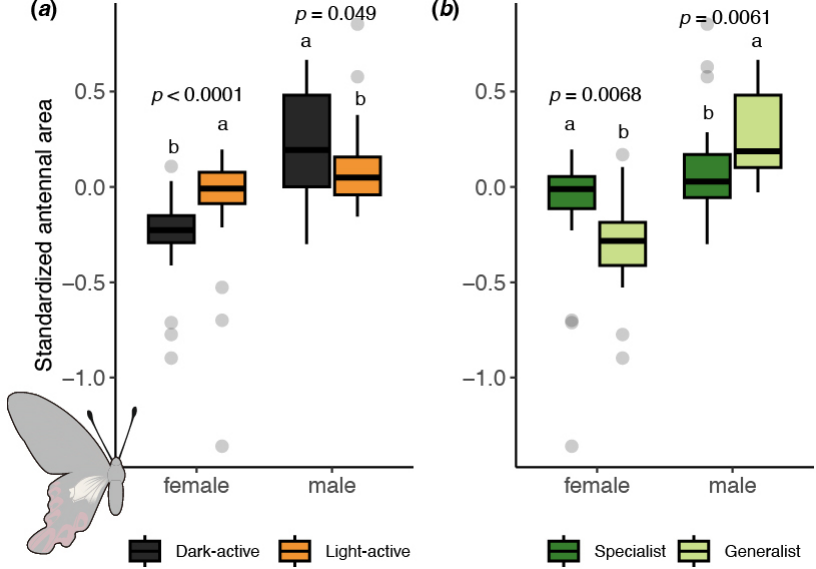
Antennal traits in moths and butterflies vary by diel activity and host specificity. Among Lepidoptera, females of light-active species have significantly larger relative antennae than those of dark-active species. (*a*) Antennal area relative to body size differs between diel activity groups (*n* = 101 light-active, *n* = 86 dark-active). (*b*) Relative antennal area also varies by host specificity (*n* = 111 specialists, *n* = 64 generalists). Different letters indicate statistical differences (Wilcoxon sum rank test) within each sex. Relative antennal areas account for body size and phylogenetic association.

We measured antennal and body size in 582 specimens from 94 species (electronic supplementary material, table S4) and found that antennal size varies with sex, diel activity and host diversity (figure 5). Females of light-active species have significantly larger relative antennal sizes than dark-active species (Wilcoxon test, *W* = 437, *p* < 0.0001), whereas males of light-active species have smaller relative antennae (*W* = 189, *p* = 0.049). Similarly, specialist females have significantly larger relative antennae than generalists (*W* = 305, *p* = 0.006), while specialist males have smaller relative antennae than generalists (*W* = 117, *p* = 0.006).

## 4. Discussion

Host plant specialization studies in the Lepidoptera have contributed much to our current understanding of coevolution and ecological specialization in general [78–82]. Numerous studies have explored the role of non-volatile plant metabolites in shaping host plant specialization patterns [12,83]. However, the potential role of plant volatiles in the evolution of host breadth remains poorly understood. Our findings suggest a potential role of plant volatile availability in influencing the evolution of host specialization of butterflies and moths. The significant correlation between lepidopteran host plant specificity and adult diel activity closely aligns with the general diel cycle of plant volatile emissions, supporting the predictions of the SAH. Specifically, we show that Lepidoptera that are active during the day (or photophase) are exposed to a greater abundance and diversity of host plant-associated volatiles. They are more likely to have specialized dietary preferences. In contrast, species active only at night (i.e. during the scotophase) encounter significantly fewer and less abundant plant volatiles. They are also less likely to specialize in particular groups of host plants.

Additionally, we found that nocturnal adult females, regardless of their degree of host specialization, tend to have smaller antennae relative to their body sizes compared with their diurnal counterparts. This suggests that the opportunity to utilize chemical information is more limited at night, leading to reduced investment in olfactory structures, which indirectly supports a key premise of the SAH. The observed differences in antennal size and diel activity patterns between sexes raise intriguing questions for future research. For instance, the smaller antennal size in nocturnal females might indicate differing sensory priorities, such as a reduced reliance on olfactory cues for activities like host location, foraging or mate finding. In contrast, the trend observed in males suggests that their increased antennal size may be influenced by different ecological pressures, such as the need to locate mates or forage efficiently. The larger antennae in generalist males may be an outcome of the scarce and unpredictable distribution of females in the environment due to the absence of a specific host plant. This unpredictability may drive a greater reliance on chemical cues for mate detection. These speculations still require further investigation, and future studies could also explore whether antennal differences correlate with other ecological or behavioural traits, such as adult foraging preferences (e.g. specialist floral visitors potentially having larger antennae). Investigating these relationships could shed light on the selective pressures driving the evolution of sensory structures in Lepidoptera.

Landscape-level studies on plant volatiles suggest that lower availability of plant volatiles at night may be a common characteristic of terrestrial ecosystems (figure 2*b*). However, exceptions exist at the individual plant level. While the majority of land plants exhibit increased volatile emissions during daylight hours—correlated with factors like light availability [84], photosynthesis [32], stomatal behaviour [85] and temperature [86]—certain plants instead release floral volatiles primarily at night [87]. Numerous studies, including those on model systems like *Petunia* spp. and *Nicotiana* spp., have highlighted these nocturnal emissions (figures 2*b* and 1*c*). Nocturnal pollination is a widespread phenomenon across angiosperms [88], with many night-blooming flowers emitting strong volatiles to attract nocturnal pollinators [88–91], which often feed on the same plant in their larval stages [92–94]. This suggests that plants emitting significant volatiles at night may be more noticeable (salient) to nocturnal herbivores, potentially facilitating host plant specialization among nocturnal Lepidoptera that co-occur with these plants.

An interesting observation is that these nocturnal-specialist Lepidoptera often associate with plants that release volatiles at night, such as the specialist tobacco hornworm moths, *Manduca sexta*, and their host plant tobacco [95]. In our analysis, the higher emission of volatiles at night is more commonly observed in herbaceous plants (figure 2c). Apart from the reasonable explanation that mutualism with nocturnal pollinators may be more common in herbaceous plants than other plant types, the number of studies focusing on specific herbaceous model systems may also influence these results (electronic supplementary material, table S5). For instance, *Nicotiana* spp. and *Petunia* spp. are known to have flowers that attract nocturnal pollinators [95,96] that are also specialist herbivores (e.g. tobacco hornworm, *Manduca sexta* [21]). Another well-documented case is the interaction between the evergreen shrub/tree genus Yucca and its specialist nocturnal herbivorous pollinator, *Tegeticula yuccasella* [92]. These nocturnal specialists could potentially serve as the ‘exception that proves the rule’ for the SAH and require further investigation.

## 5. Considerations for future studies

While our findings support the SAH, several limitations must be acknowledged to refine future research. A key constraint is the absence of a well-resolved Lepidoptera phylogeny for comparative analysis [64]. Although we sampled about half of Lepidoptera families, this represents less than 0.1% of species diversity. Early branching clades like Micropterigoidea, Agathiphagoidea and Heterobathmioidea are absent, and their life histories remain poorly known. Additionally, we undersampled speciose families such as Noctuidae, Crambidae and Pyralidae, particularly diurnal species. These gaps stem from phylogenetic and trait data constraints rather than deliberate exclusions. For instance, classifying Mimallonoidea as diurnal contradicts the common view that most mimallonids are nocturnal, underscoring the need for better sampling. Similarly, Hedylidae, a nocturnal family within diurnal Papilionoidea, was omitted due to data limitations, impeding tests of SAH predictions about nocturnality reversals and associated traits like polyphagy and antennal morphology.

Underrepresentation of clades with variable diel activity, such as Erebidae and Geometridae, limits the generalizability of our findings. Seifert *et al*. [97] found only a marginal link between diurnality and host specialization in Geometridae, aligning with SAH but highlighting the need for broader datasets and lineage-specific analyses. Furthermore, our antennal morphology analysis, constrained by uneven taxonomic coverage, relies on existing phylogenies and available specimens. These limitations emphasize the need for expanded phylogenetic and morphological data to refine future studies.

Limited taxon sampling also affects ancestral state reconstructions. Expanding coverage to include early-branching groups, Hedylidae, and diurnal species in Noctuidae and Pyralidae is critical for more reliable analyses. Future research should leverage museum specimens, enhance phylogenetic data and integrate detailed life-history information to fill these gaps. Addressing these challenges will clarify how diel activity, host specialization and antennal morphology are shaped by ecological factors like host plant volatiles, advancing our understanding of Lepidopteran specialization.

## 6. Conclusion

Recent advances in chemical ecology have deepened our understanding of how plant volatiles influence herbivore behaviour and evolution. The SAH offers a mechanistic framework linking plant volatile availability to host specialization in Lepidoptera. Based on plant volatile emissions, Lepidoptera diel and diet preferences, and antennal morphology, our study provides three lines of evidence supporting the SAH while offering novel insights into herbivore navigation of complex ecological landscapes. However, taxon sampling limitations warrant cautious interpretation. Despite these constraints, the SAH has significant potential for elucidating how plant volatiles drive host specialization in Lepidoptera and other herbivorous lineages.

Deviations from SAH predictions present intriguing cases for further study, helping to uncover mechanisms underlying ecological specialization and generalization. Future research should improve sampling of underrepresented Lepidoptera families and explore diel activity as a labile trait in Geometridae, Noctuidae and Pyralidae. Species-level studies will further clarify connections between diel activity, sensory adaptations and host plant specialization. Additionally, the SAH offers opportunities to investigate multi-modal plant–insect interactions, integrating sensory modalities like visual and tactile cues. Addressing current limitations and adopting a multidisciplinary approach will refine hypothesis testing, revealing new dimensions of plant–insect interactions and ecological specialization.

## Data Availability

The data supporting the findings of this study are included as supplementary information accompanying this publication. Supplementary material is available online [98].
